# Genome-Wide Identification and Analysis of Phospholipase C Gene Family Reveals Orthologs, Co-Expression Networks, and Expression Profiling Under Abiotic Stress in *Sorghum bicolor*

**DOI:** 10.3390/plants13212976

**Published:** 2024-10-24

**Authors:** Hongcheng Wang, Junxing Yu, Xingyu Zhang, Qian Zeng, Tuo Zeng, Lei Gu, Bin Zhu, Feng Yu, Xuye Du

**Affiliations:** 1 School of Life Sciences, Guizhou Normal University, Guiyang 550025, China; wanghc@gznu.edu.cn (H.W.); 221109030181@gznu.edu.cn (Q.Z.); zengtuo@gznu.edu.cn (T.Z.); 201808009@gznu.edu.cn (L.G.); 201703008@gznu.edu.cn (B.Z.); 2 School of Energy and Environment, City University of Hong Kong, Hong Kong 999077, China; xzhang3864-c@my.cityu.edu.hk

**Keywords:** sorghum, PLC, abiotic stress, WGCNA, expression analysis

## Abstract

Phospholipase C (PLC) is an essential enzyme involved in lipid signaling pathways crucial for regulating plant growth and responding to environmental stress. In sorghum, 11 PLC genes have been identified, comprising 6 *PI-PLCs* and 5 *NPCs*. Through phylogenetic and interspecies collinearity analyses, structural similarities between SbPLCs and ZmPLCs proteins have been observed, with a particularly strong collinearity between SbPLCs and OsPLCs. Promoter function analysis has shown that *SbPLCs* are significantly enriched under abiotic stress and hormonal stimuli, like ABA, jasmonic acid, drought, high temperature, and salt. Gene co-expression networks, constructed using a weighted gene co-expression network analysis (WGCNA), highlight distinct expression patterns of *SbPLC1*, *SbPLC3a*, and *SbPLC4* in response to abiotic stress, providing further insights into the expression patterns and interactions of *SbPLCs* under various environmental stimuli. qRT-PCR results reveal variations in expression levels among most *SbPLCs* members under different stress conditions (drought, NaCl, NaHCO_3_), hormone treatments (ABA), and developmental stages, indicating both specific and overlapping expression patterns. This comprehensive analysis offers valuable insights into the roles of *SbPLCs* in sorghum, shedding light on their specific expression patterns, regulatory elements, and protein interactions across different environmental stimuli and developmental stages.

## 1. Introduction

Phospholipases, such as Phospholipase A1 (PLA1), Phospholipase A2 (PLA2), Phospholipase B (PLB), Phospholipase C (PLC), and Phospholipase D (PLD), are essential enzymes with diverse physiological functions in organisms [1]. They are involved in cellular metabolism [2], signal transduction [3], immune responses [4,5], and various biological processes. Among these, PLC plays a significant role in lipid signaling and is crucial for plant life activities. PLC can be further classified into Phosphatidylinositol-specific Phospholipase C (PI-PLCs) and Phosphatidylcholine Phospholipase C (PC-PLC), also known as Non-Specific Phospholipase C (NPC), based on its substrates [6,7].

Phospholipase C facilitates the breakdown of phosphatidylinositol 4,5-bisphosphate (PIP2) into inositol 1,4,5-trisphosphate (IP3) and diacylglycerol (DAG) [8,9], which are crucial secondary messengers. This process leads to the release of Ca^2+^ from the cell, activating members of the protein kinase C (PKC) family [10]. Plant PI-PLC enzymes typically consist of two important domains: the catalytic PI-PLCX domain and the PI-PLC-Y domain [11], both of which are necessary for PI-PLC activity [12,13]. Additionally, PI-PLCs contain a C-terminal Ca^2+^/phospholipid-binding C2 domain and an N-terminal EF-hand domain that are involved in calcium binding [14]. Some plant NPCs have putative signal peptides at the N-terminus, while all NPCs share a phosphodiesterase domain that is crucial for esterase functionality [8].

As an essential component of lipid signaling, PLC plays a crucial role in plant growth and development and has been identified in various plant species. This includes 9 PI-PLC and 6 NPC genes in *Arabidopsis thaliana*, 4 PI-PLC and 5 NPC genes in rice (*Oryza sativa* L.) [15], 5 PI-PLC and 4 NPC genes in maize (*Zea mays* L.) [16], and 7 PI-PLC and 3 NPC genes in tomato (*Solanum lycopersicum* L.) [17]. Research has shown that PI-PLC is involved not only in plant responses to a variety of stresses, such as osmotic, heat, cold, and heavy metal stress, but it also plays key roles in numerous physiological processes [18,19]. During osmotic stress, PLC helps regulate the generation of reactive oxygen species (ROS), microtubule dynamics, and the accumulation of metabolites such as proline [20,21]. Additionally, PLC enhances salt tolerance in *Arabidopsis* by modulating Ca^2^⁺ signaling in seedlings [22]. Under heat stress, PLC participates in the activation of heat shock protein expression, and the overexpression of *AtPLC9* increases heat tolerance in *Arabidopsis* [23,24]. These studies indicate that PLC plays a central role in signal transduction and adaptive mechanisms under various environmental stress conditions. Moreover, PLC is critical for plant growth and development. In *Arabidopsis*, PLC is involved in ABA-mediated seed germination and seedling growth [25,26], while in potato, PLC facilitates tuber differentiation through IP3 generation. Additionally, PLC is essential for photosynthesis and the growth and development of maize seedlings [27].

Sorghum (*Sorghum bicolor* L.) is a versatile crop with high yield potential and potential applications in energy production, human consumption, animal feed, and industrial uses. This plant shows promise in adapting to various environmental stresses like drought, salinity, and high temperatures [28]. Recent advancements in molecular and systems biology tools have opened up new avenues for breeders to enhance stress resistance in sorghum [29,30]. Despite this progress, there is a lack of comprehensive information on the functional roles of the *SbPLCs* gene family across developmental stages and stress conditions. This study aims to fill this gap by conducting a detailed analysis of the *SbPLCs* gene family at a whole-genome level, exploring their evolutionary patterns, protein structures, and expression profiles in different tissues and stress environments. The findings shed light on the significance of *SbPLCs* in growth, development, and stress responses, offering valuable genetic resources for breeding resilient crops.

## 2. Results

### 2.1. Identification and Physicochemical Property Analysis of the SbPLCs Family

Utilizing the Hidden Markov Model (HMM) method and BLASTP (protein blast), a total of 11 SbPLCs were identified. The *SbPLCs* family can be categorized into two subfamilies, *SbPI-PLCs* and *SbNPCs*, based on gene structure, protein domains, and evolutionary relationships. The *SbPI-PLCs* subfamily includes six members named *SbPI-PLC1*/*2*/*3a*/*3b*/*5*, while the *SbNPCs* subfamily comprises five members named *SbNPC1*/*2*/*3*/*4*/*5* (Table 1). The amino acid count of SbPI-PLC proteins ranges from 607 to 627 amino acids (AA), with molecular weights varying from 67.50 to 77.76 kDa. Their isoelectric points (IP) are predominantly below 7, indicating a mildly acidic nature, except for SbPI-PLC5, which is neutral at 7.13. The hydrophilicity coefficient for all PI-PLCs proteins is below 0, suggesting their hydrophilic nature. In contrast, for SbNPC proteins, the amino acid count ranges from 510 to 578 AA, with molecular weights between 57.67 and 63.43 kDa. Notably, SbNPC1 and SbNPC5 exhibit isoelectric points of 7.18 and 9.17, respectively, while the remaining proteins have isoelectric points below 7. The hydrophilicity coefficient for all NPC proteins is also below 0. Subcellular localization prediction indicates that SbPI-PLC proteins are likely to be found in the cytoplasm or on the cell membrane, with only SbPI-PLC5 predicted to be localized in the cytoplasm. On the other hand, SbNPC proteins are predicted to be localized in the chloroplast or mitochondria.

### 2.2. Evolutionary Relationship Analysis of SbPLCs

**Figure 1:**
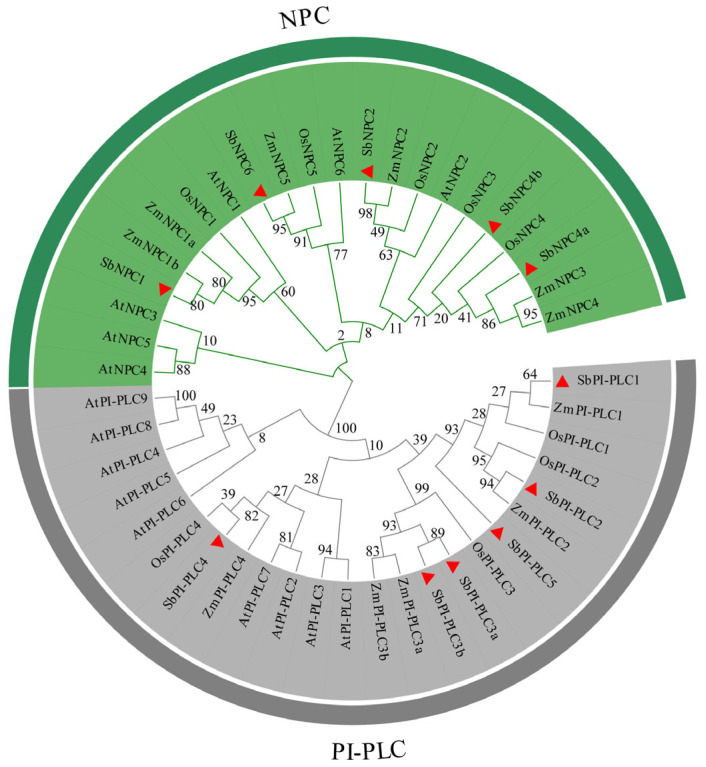
Phylogenetic analysis of the PLC family. The green section indicates the NPC subfamily, and the gray section indicates the PI-PLC subfamily. The analysis involved 46 amino acid sequences from various plants, including *Arabidopsis thaliana* (At), *Oryza sativa* (Os), *Sorghum bicolor* (Sb), and *Zea mays* (Zm).

To investigate the evolutionary relationships of PLCs between sorghum and other species, we analyzed 15 AtPLCs from *A. thaliana*, 9 OsPLCs from *O. sativa*, 11 ZmPLCs from *Z. mays*, and 11 SbPLCs from sorghum. A maximum likelihood (ML) phylogenetic tree was generated (Figure 1). Through sequence alignment and phylogenetic analysis, the 46 proteins were categorized into two subfamilies, PI-PLCs and NPC. SbPLCs proteins show a close association with ZmPLCs proteins, suggesting a strong relationship, but they are more distantly related to AtPLCs proteins.

### 2.3. Analysis of Gene Structure and Motif Sequences of SbPLCs

**Figure 2:**
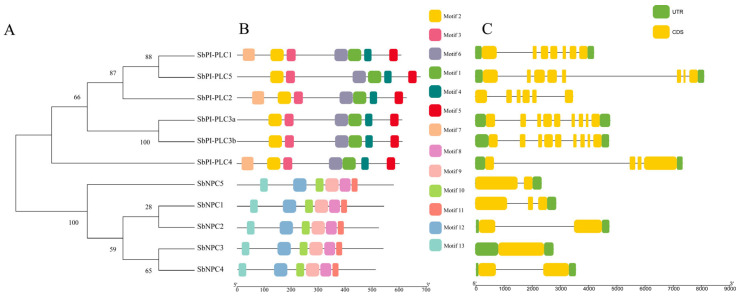
Conserved motifs and exon–intron structure analysis of the PLC gene in sorghum. (**A**) Phylogenetic tree of SbPLCs. The tree was generated using MEGA7 through the maximum likelihood method based on the protein sequences of SbPLCs. (**B**) Conserved motif analysis. Eleven motifs were identified, with different colored boxes representing various types of motifs. (**C**) Exon–intron structures. Exons and introns of *S. bicolor* are depicted as yellow rounded rectangles and thin lines.

There is a notable difference in the gene structure of *SbPI-PLCs* and *SbNPCs*. *SbPI-PLCs* exhibited a larger number of exons and introns, with a minimum of four and a maximum of nine exons, and a minimum of three and a maximum of seven introns. Additionally, *SbPI-PLC2* contains a UTR region. On the other hand, *SbNPCs* generally have one to three exons, with *SbNPC1* having two introns, *SbNPC3* lacking an intron, and the rest having only one intron (Figure 2C).

Conserved motifs in proteins play a crucial role in forming their functional domains. *PI-PLCs* possess four major functional domains: the EF-hand-like domain, the PI-PLC-X domain, the PI-PLC-Y domain, and the C2 domain. Motif 7 constitutes the main part of the EF-hand-like domain, motifs 2 and 3 contribute to the PI-PLC-X domain, motifs 6 and 1 contribute to the PI-PLC-Y domain, and motifs 4 and 5 constitute the main parts of the C2 domain. Motifs 8, 9, 10, 11, 12, and 13 collectively form the phosphodiesterase domain of NPC (Figure 2B). Among them, only *SbPLC1*, *SbPLC2*, and *SbPLC4* have the EF-hand-like domain, while *SbPLC3a*, *SbPLC3b*, and *SbPLC5* lack the EF-hand-like domain. The position markers for these domains are indicated in (Figure S1).

### 2.4. Chromosomal Localization and Gene Replacement Rates of SbPLCs Genes

**Figure 3:**
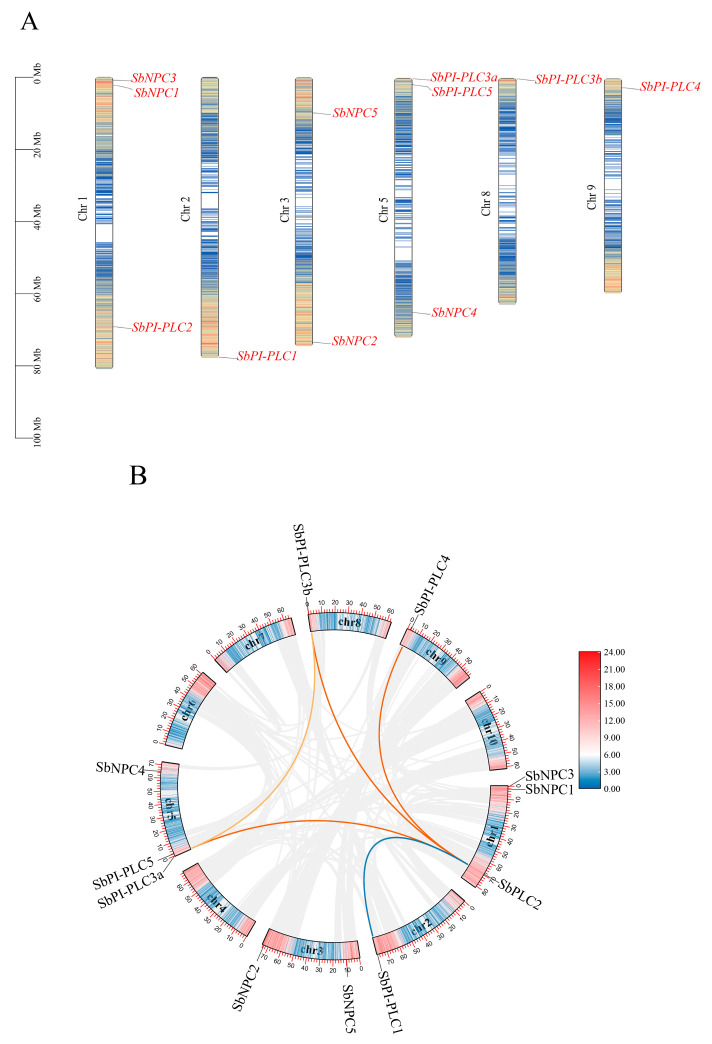
Chromosomal mapping, gene replication, and synteny analysis of *SbPLCs*. (**A**) Chromosomal localization of *SbPLCs*. The yellow font indicates the sorghum chromosome number, the red font displays the names of the *SbPLCs*, and the scale on the left represents the length of each chromosome. (**B**) Gene replication and synteny analysis of *SbPLCs*. The outer box represents the GC content of the chromosome, while the inner box illustrates the gene density of the chromosome. The names of the *SbPLCs* genes are highlighted.

The distribution of the 11 *SbPLCs* genes spans 5 chromosomes. Chromosome 1 hosts *SbPI-PLC1*, *SbNPC3*, and *SbNPC1*. Chromosome 5 contains *SbPI-PLC3a*, *SbPI-PLC5*, and *SbNPC4*. *SbNPC2* and *SbNPC5* are located on chromosome 3, while *SbPI-PLC1* is situated on chromosome 2. *SbPI-PLC3b* resides on chromosome 4, and *SbPI-PLC4* is situated on chromosome 9 (Figure 3A).

The Ka/Ks ratio serves as an indicator of selective pressures between genes. In the case of *SbPLCs*, the Ka/Ks values between *SbPI-PLCs* and *SbNPCs* are close to 1, suggesting that these genes have maintained conservative functions throughout evolution. Interestingly, *SbPI-PLC2* with *SbPI-PLC4* and *SbNPC3* with *SbNPC4* show Ka/Ks values higher than one, indicating positive selection possibly due to their involvement in distinct functions over evolutionary time (Table S2).

### 2.5. Gene Duplication Events and Synteny Analysis of SbPLCs

Gene duplication plays a crucial role in species evolution, with segmental and tandem duplications being considered the two main mechanisms for expanding gene families [31]. An analysis of gene duplications in *SbPLCs* revealed no tandem duplications among *SbPI-PLCs*, and all duplications occurred through segmental duplication, suggesting that the expansion of the *SbPI-PLCs* family primarily occurred through segmental duplication. No gene duplications were detected among *SbNPCs* (Figure 3B).

**Figure 4:**
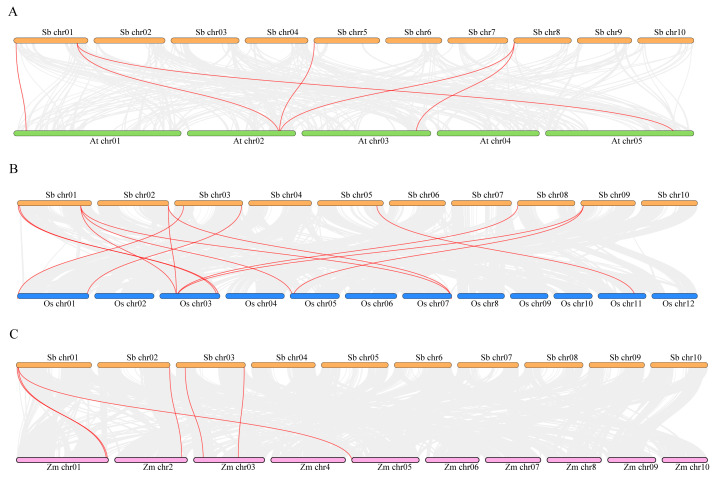
Collinearity analysis of the PLC gene in sorghum with other species. (**A**) Synteny analysis of *SbPLCs* and *AtPLCs*. (**B**) Synteny analysis of *SbPLCs* and *ZmPLCs*. (**C**) Synteny analysis of *SbPLCs* and *OsPLCs*. The red lines highlight the syntenic PLC gene pairs.

To understand the homologous relationships between *PLC* genes in sorghum and other plants, synteny analysis was performed with *A. thaliana*, *O. sativa*, and *Z. may*. Six pairs of syntenic gene pairs were identified in *A. thaliana* (Figure 4A), six in *Z. may* (Figure 4B), and thirteen in *O. sativa* (Figure 4C). Among these, only one pair was syntenic with *A. thaliana* and involved *NPC* genes, while only one pair was syntenic with *Z. may* and involved *PI-PLC* genes (Table S3).

### 2.6. Analysis of Cis-Acting Elements in SbPLCs Promoters

**Figure 5:**
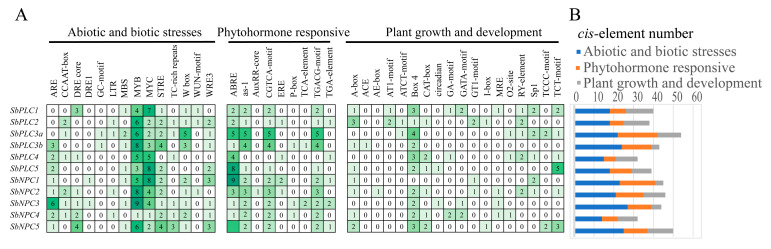
*Cis*-element number analysis in the *SbPLCs* gene family. (**A**) The different intensity of colors and numbers of the grid indicate the numbers of different promoter elements in the *SbPLCs* genes. (**B**) The different colored histogram represents the sum of the cis-acting elements in each category.

To understand the expression patterns and potential functions of *SbPLCs* under different conditions, the 2000 base pairs upstream of the translation initiation site for each gene were submitted to the Plant CARE database to retrieve putative cis-acting elements. The identified elements were categorized into three groups: abiotic and biotic stresses elements, phytohormone responsive elements, and plant growth and development elements (Figure 5).

The category with the highest number of elements for each gene was abiotic and biotic stress, with *SbNPC4* having the least (14 elements) and *SbNPC3* having the most (27 elements). The elements MYB and MYC were the most abundant, suggesting that *SbPLCs* might be regulated by MYB and MYC transcription factors, thereby expressing under plant stress. In the phytohormone responsive category, ABRE elements were present in the promoter regions of each gene, indicating that *SbPLCs* might be regulated by the ABA hormone.

### 2.7. Prediction of Transcription Factors Modulating SbPLCs

**Figure 6:**
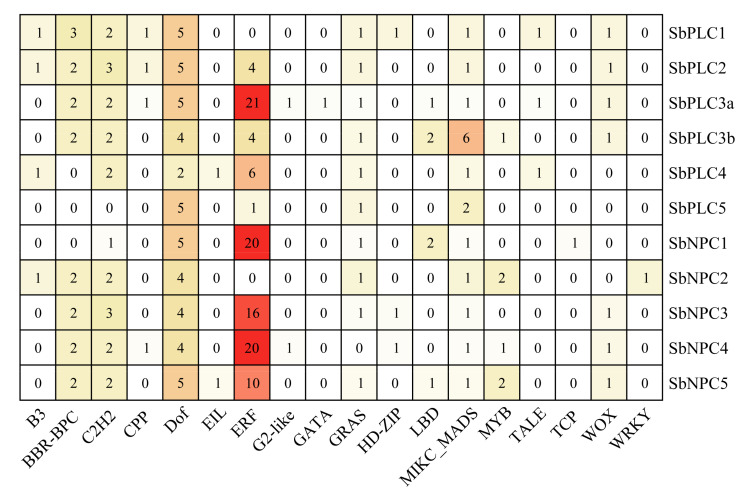
Prediction of transcription factors regulating *SbPLCs* in sorghum. The numbers and varying intensity colors within the grid represent the quantity of different transcription factors within the *SbPLCs*.

TFs play a crucial role in governing gene expression in plant biology. After prediction using PlantRegMap, we discerned a total of 18 TF families comprising 59 TFs (Figure 6 and Table S5). Among these families, MIKC-type MADS-box (MIKC_MADS), DNA-binding with one finger (Dof), and Ethylene Response Factor (ERF) were the most prevalent, with 6, 5, and 23 TFs, respectively. Notably, six MIKC_MADS TFs were identified in SbPLC3b, while all SbPLCs harbored Dof TFs, with nearly all sharing these five TFs except for SbPLC4, which had only two Dof TFs. Although ERF TFs were the most abundant, *SbPLC1* and *SbNPC2* lacked them, and only 1 ERF TF was found in *SbPLC5*. *SbPLC3a, SbNPC1, SbNPC3*, and *SbNPC4* were associated with 21, 20, 16, and 20 TFs, respectively. The involvement of ERF genes in modulating plant growth and responses to diverse stressors underscores the significant role of *SbPLCs* in sorghum’s resilience to abiotic stressors.

### 2.8. miRNA Targeting Analysis of SbPLCs at the Whole Genome Level

**Figure 7:**
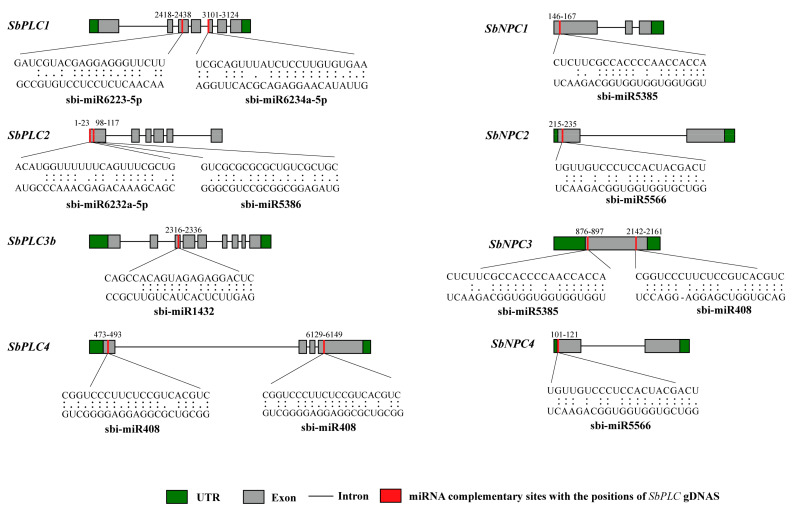
miRNA targeting *SbPLCs*. Schematic illustration indicates the *SbPLCs* targeted by miRNAs. The RNA sequence of each complementary site is from 5′–3′, and the predicted miRNA sequence from 3′–5′ is exposed in the long-drawn-out areas. See Table S5 for the detailed data of all predicted miRNAs.

Recent studies have indicated the involvement of miRNA-mediated regulation in plant stress responses. To further understand the post-transcriptional regulation of *SbPLCs* s by miRNAs, we predicted 9 miRNAs targeting 8 genes (Figure 7; Table S5). Figure 7 depicts the miRNA targeting sites, while detailed information on all miRNA-targeted genes/sites is provided in Table S5. Our findings revealed that sbi-miR408 targets two genes (*SbPLC4* and *SbNPC3*), sbi-miR5385 targets two genes (*SbNPC1* and *SbNPC3*), sbi-miR5566 targets two genes (*SbNPC2* and *SbNPC4*), sbi-miR6223 targets two genes (*SbPLC1* and *SbPLC5*), and two members of the sbi-miR6232 family target two genes each (*SbPLC2* and *SbPLC5*). Additionally, sbi-miR397 and sbi-miR6229 target one gene each (*SbPLC5*), while sbi-miR6234a and sbi-miR1432 target one gene each (*SbPLC1* and *SbPLC3b*) (Figure 7; Table S5). The expression levels of these miRNAs and their targeted genes require validation in further studies to elucidate their biological roles in the sorghum genome.

### 2.9. Expression Patterns of SbPLCs in Different Tissues of Sorghum

**Figure 8:**
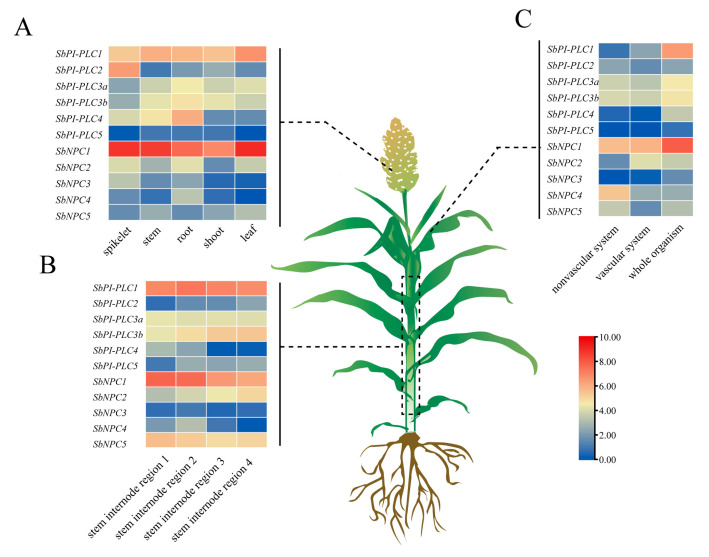
The expression profile of *SbPLCs* in different tissues and developmental stages. (**A**) Expression levels in major biological tissues. (**B**) Expression levels in vascular systems. (**C**) Expression levels in biomass accumulation. The sorghum was created using BioRender (https://biorender.com/ (accessed on 15 June 2024)).

The expression patterns of 11 *SbPLCs* in various tissues and organs were analyzed using the Sorghum Expression Database (SorghumFDB), and a heatmap was constructed (Figure 8). Expression patterns of *SbPI-PLCs* and *SbNPCs* were examined across diverse tissues. *SbPI-PLC1* showed substantial expression in leaves, roots, and spikelets, whereas *SbPI-PLC2* exhibited higher expression in stems. Notably, *SbNPC1* displayed the highest expression in leaves and spikelets, emphasizing its crucial role in these tissues (Figure 8A). In stem internode regions (Figure 8B), *SbPI-PLC1* and *SbNPC1* demonstrated varied expression, with higher levels in regions one and two. In contrast, *SbPI-PLC2* displayed increased expression in regions three and four, suggesting region-specific functions in stem development. The expression levels in nonvascular systems, vascular systems, and whole organisms are depicted (Figure 8C). *SbPI-PLC1* exhibited notable expression in whole organisms and vascular systems, while *SbPI-PLC2* showed increased expression in nonvascular systems and whole organisms. *SbNPC1* displayed substantial expression in whole organisms and vascular systems, highlighting its significance in these biological contexts. These findings provide insights into the tissue-specific expression patterns of *SbPI-PLC* and *SbNPC*, contributing to our understanding of their roles in sorghum development.

### 2.10. Expression Profiles of SbPLC Genes Under Abiotic Stress

**Figure 9:**
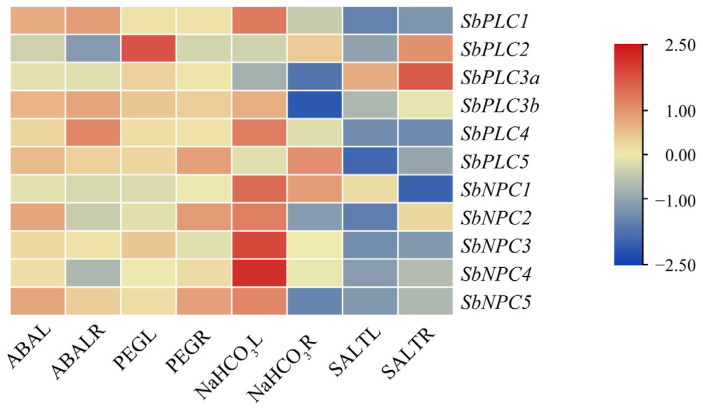
Expression heatmap of *SbPLCs* under abiotic stress. The heatmap depicts the expression levels of *SbPLCs* in response to different abiotic stressors, which were analyzed and visualized using TBtools. Each row represents a gene, and each column represents a specific stress condition or time point. The color intensity reflects the relative expression level, with warmer colors indicating higher expression and cooler colors indicating lower expression. All transcriptional expression data were derived from transcriptome analysis.

To explore the expression patterns of *SbPLCs* under abiotic stress, transcriptome data from sorghum were analyzed, selecting data from four different conditions (Figure 9). It was found that *SbPLCs* exhibit distinct expression patterns under different abiotic stress conditions. The most noticeable changes were observed under NaHCO_3_ and salt stress. Specifically, in the aerial parts after NaHCO_3_ treatment, except for *SbPLC5*, the expression levels of all other *SbPLCs* varied. *SbPLC2* and *SbPLC3a* showed a downward trend, while the remaining eight family members were upregulated, with *SbNPC3* and *SbNPC4* showing the highest upregulation. Conversely, in the underground parts after NaHCO_3_ treatment, *SbNPC3* and *SbNPC4* showed no significant changes, while a significant downward trend was observed for *SbPLC3a* and *SbPLC3b*. Under salt stress, almost all *SbPLCs* exhibited a downward trend in both aerial and underground parts, with only *SbPLC3a* and *SbNPC1* showing an upward trend in the aerial parts, and *SbPLC2*, *SbPLC3a*, and *SbNPC2* showing an upward trend in the underground parts. *SbPLC3b* showed no significant changes in expression. These results indicate that *SbPLCs* play diverse roles in sorghum’s response to abiotic stressors.

### 2.11. Construction of the Co-Expression Network and Protein–Protein Interaction Network

**Figure 10:**
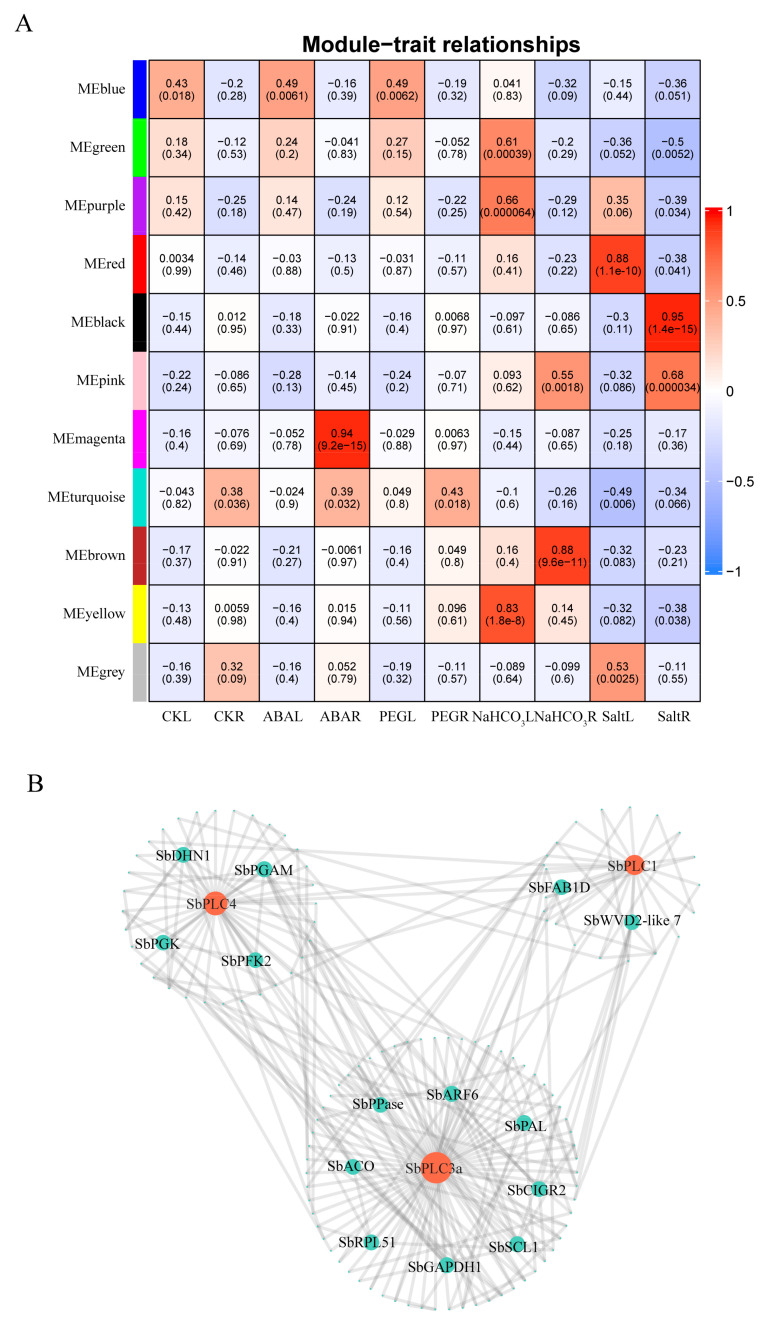
Co-expression network analysis of *SbPLCs*. (**A**) Weighted gene co-expression network analysis (WGCNA) of SbPLCs genes. The heatmap displays Pearson correlations between module eigengenes (MEs) and treatments, with red and blue color scales on the right indicating positive and negative correlations, respectively. Each row corresponds to a module. Cells show correlation coefficients (above) and corresponding *p*-values (below), with coefficients ≥ 0.8 and *p*-values ≤ 0.05 considered significant. (**B**) Protein–protein interaction (PPI) network composed of filtered *SbPLCs* co-expression genes. Node size represents degree centrality. All transcriptional expression data were derived from transcriptome analysis.

To identify important regulatory genes and the regulatory roles of genes under abiotic stress, we constructed a gene co-expression network using WGCNA. A total of 11 modules were constructed, correlating with 10 phenotypic data (CKL, CKR, ABAL, ABAR, PEGL, PEGR, NaHCO_3_L, NaHCO_3_R, SALTL, SALTR), determined based on a *p*-value < 0.05 threshold, resulting in 20 treatment/organ-related modules (Figure 10A). Among these, five modules exhibited a strong positive correlation with phenotypic features, with correlation coefficients ≥ 0.8 and *p*-value ≤ 0.05, namely: ABAR/magenta (0.94), NaHCO_3_L/yellow (0.83), NaHCO_3_R/brown (0.88), SALTL/red (0.88), and SALTR/black (0.95). Within these correlated modules, we found three modules containing *SbPLCs*, i.e., ABAR/magenta containing *SbPLC4*, NaHCO_3_L/yellow containing *SbPLC1*, and SALTR/black containing *SbPLC3a*.

To further investigate the response of *SbPLCs* to abiotic stress within specific modules, we conducted an analysis on the weighted correlation of *SbPLC1*, *SbPLC3a*, and *SbPLC4* with other genes. Our findings indicated that using a threshold of weighted correlation ≥ 0.2, the ABAR/magenta module exhibited 94 co-expressed genes with *SbPLC4*, the NaHCO_3_L/yellow module showed 52 co-expressed genes with *SbPLC1*, and the SALTR/black module displayed 179 co-expressed genes with *SbPLC3a*. Particularly noteworthy was the higher number of co-expressed genes with *SbPLC3a* in the SALTR/black module, suggesting its significant role in salt stress response.

Additionally, A PPI network was created involving the three genes mentioned (*SbPLC1*, *SbPLC3a*, and *SbPLC4*) and their co-expressed genes, which revealed interactions with 14 proteins (Figure 10B), i.e., (i) SbFAB1D (SORBI_3003G373500) and SbWVD2-like 7 (SORBI_3003G380500) are associated with SbPLC1, (ii) SbARF6 (SORBI_3004G051900), SbPAL (SORBI_3004G220400), SbCIGR2 (SORBI_3002G354900), SbSCL1 (SORBI_3009G143400), SbGAPDH1 (SORBI_3010G262500), SbRPL51 (SORBI_3001G462900), SbACO (SORBI_3002G316900), and SbPPase (SORBI_3009G152600) are associated with SbPLC3a, and (iii) SbDHN1 (SORBI_3009G116700), SbPGAM (SORBI_3001G384100), SbPFK2 (SORBI_3006G114700), and SbPGK (SORBI_3010G221800) are associated with SbPLC4. It is plausible that genes encoding proteins with higher connectivity to *SbPLC1*, *SbPLC3a*, and *SbPLC4* also play a crucial role in responding to abiotic stress.

### 2.12. GO and KEGG Analysis of Co-Expressed Genes

**Figure 11:**
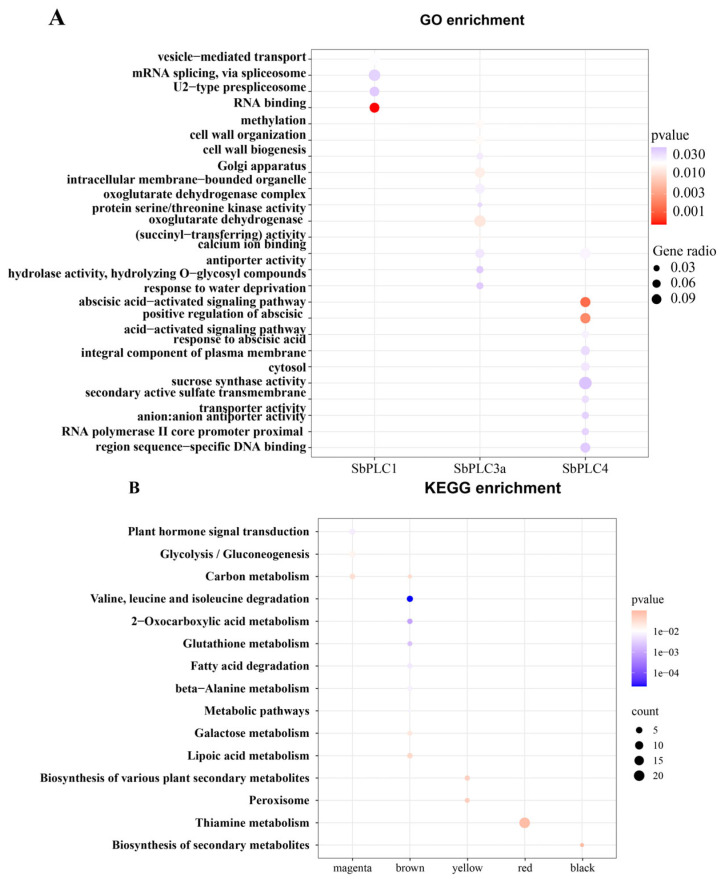
The GO and KEGG enrichment analysis of SbPLCs genes. (**A**) Heatmap of GO enrichment analysis for co-expression genes of *SbPLCs*. (**B**) KEGG conducted significant enrichment analysis on genes from five significantly co-expressed modules.

To explore the functions of *SbPLC1*, *SbPLC3a*, and *SbPLC4*, along with their co-expressed genes, GO annotation and analysis were conducted (Figure 11A). The results revealed that the GO enrichment of the *SbPLC1* gene and its co-expressed genes included (i) mRNA processing (GO:0006397), vesicle-mediated transport (GO:0016192), mRNA splicing via spliceosome (GO:0000398), (ii) U2-type prespliceosome (GO:0071004) in the “cellular components”, and (iii) RNA binding (GO:0003723) in the “molecular functions”. These GO terms mainly relate to mRNA transport processing, a crucial part of gene expression regulation, suggesting a potential role of *SbPLC1* in mRNA processing synthesis.

For the *SbPLC3a* gene and its co-expressed genes, the GO enrichment included (i) methylation (GO:0032259), cell wall organization (GO:0071555), and cell wall biogenesis (GO:0042546) in the “biological processes”, (ii) Golgi apparatus (GO:0005794), intracellular membrane-bounded organelle (GO:0043231), and oxoglutarate dehydrogenase complex (GO:0045252) in the “cellular components”, and (iii) protein serine/threonine kinase activity (GO:0004674), oxoglutarate dehydrogenase (succinyl-transferring) activity (GO:0004591), calcium ion binding (GO:0005509), antiporter activity (GO:0015297), hydrolase activity, and hydrolyzing O-glycosyl compounds (GO:0004553) in the “molecular functions”. These GO terms mainly relate to cell wall and cell membrane biosynthesis, indicating a potential association of *SbPLC3a* with protein synthesis on the cell wall.

For the *SbPLC4* gene and its co-expressed genes, the GO enrichment included (i) response to water deprivation (GO:0009414), an abscisic acid-activated signaling pathway (GO:0009738), the positive regulation of abscisic acid-activated signaling pathway (GO:0009789), and response to abscisic acid (GO:0009737) in the “biological processes”, (ii) integral component of plasma membrane (GO:0005887) and cytosol (GO:0005829) in the “cellular components”, and (iii) calcium ion binding (GO:0005509), sucrose synthase activity (GO:0016157), secondary active sulfate transmembrane transporter activity (GO:0008271), anion antiporter activity (GO:0015301), and RNA polymerase II core promoter proximal region sequence-specific DNA binding (GO:0000978) in the “molecular functions”. Most of these GO terms are associated with responses to abiotic stress and ABA hormone signaling, suggesting the involvement of *SbPLC4* in non-biological stress signal transduction.

Subsequent KEGG enrichment analysis of the three genes and their co-expressed genes revealed that *SbPLC1* and its co-expressed genes did not enrich any KEGG pathways significantly (Figure 11B). However, *SbPLC3a* was enriched in two KEGG pathways: the biosynthesis of secondary metabolites (sbi01110) and phenylpropanoid biosynthesis (sbi00940), albeit not significantly. Only *SbPLC4* was enriched in two pathways: glycolysis/gluconeogenesis (sbi00010) and carbon metabolism (sbi01200).

These findings provide insights into the functional roles of *SbPLC1*, *SbPLC3a*, and *SbPLC4* genes in sorghum, particularly in response to various environmental stimuli and their involvement in different biological processes and pathways.

### 2.13. Validation of Expression Patterns of SbPLCs Using qRT-PCR

**Figure 12:**
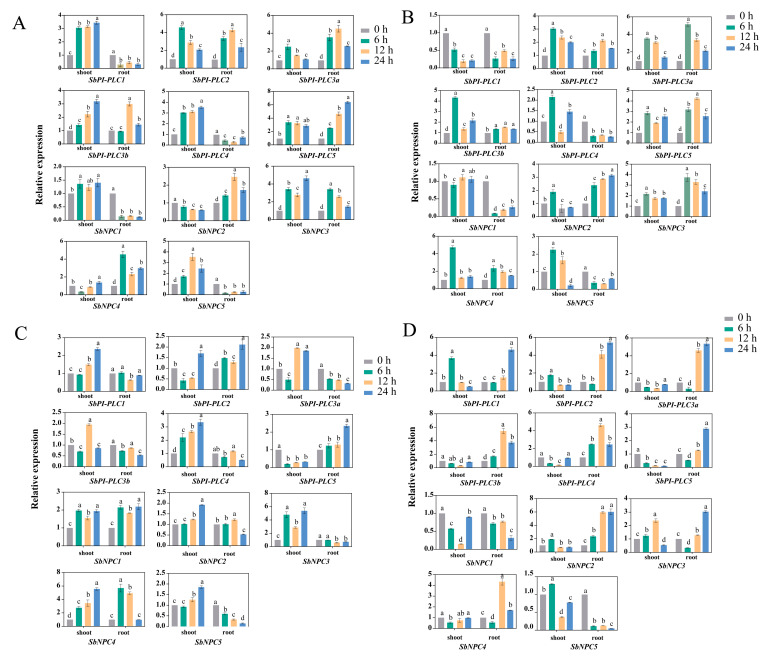
Gene expression map of *SbPLCs* under different abiotic stress. (**A**) 100 μM ABA. (**B**) Drought (20% PEG6000). (**C**) 150 mM NaHCO_3_. (**D**) 150 mM NaCl. Gene transcription levels were calculated using the 2^−ΔΔct^ method. Different letters marked on the same bar chart indicate a statistical significance of *p* < 0.05.

To investigate the response of *SbPLCs* under abiotic stress, this study selected treatments with 100 μM ABA, 20% PEG, 150 mM NaCl, and 150 mM NaHCO_3_ (pH = 8). The transcription levels of *SbPLCs* were determined using qRT-PCR at 6 h, 12 h, and 24 h after exposure to each stress. The results revealed distinct expression patterns of PLC genes following abiotic stress. Under ABA treatment, in the shoot region, all genes except *SbNPC2* showed an upregulated trend, with *SbNPC4* initially downregulated and then upregulated. In the underground parts, *SbPI-PLC1*, *SbNPC1*, and *SbNPC5* exhibited a downregulated trend (Figure 12A). Following treatment with 20% PEG, in the shoot region, *SbPI-PLC1* exhibited decreased expression, *SbNPC1* maintained a relatively stable expression, while other *SbPLCs* showed an upregulated trend. In the underground parts, the expression levels of *SbPI-PLC1*, *SbPI-PLC4*, *SbNPC1*, and *SbNPC5* decreased (Figure 12B). After NaHCO_3_ treatment, only *SbPI-PLC5* showed decreased expression in the aerial parts, while in the underground parts, the expression levels of *SbPI-PLC3a*, *SbPLC3b*, and *SbPLC6* decreased (Figure 12C). Following NaCl treatment, the aerial parts exhibited a different pattern compared to other treatments, with most genes showing a downregulated trend, except for *SbPI-PLC1*, *SbPI-PLC2*, and *SbNPC3*, which exhibited an upregulated trend. In the underground parts, the expression levels of *SbNPC1* and *SbNPC5* showed a downregulated trend (Figure 12D).

## 3. Discussion

In this study, we employed a whole genome HMM search to identify members of the *PLC* gene family in sorghum, revealing a total of 11 *PLC* genes. The six PI-PLCs in sorghum comprise three core structural domains, including PLC-X, PLC-Y, and C2, along with an EF-hand-like structure (Figure S3). In contrast, NPC possesses only one phospholipase domain. It is worth noting that in the animal kingdom, the PI-PLC family possesses two pairs of EF-hand domains capable of sensing Ca^2+^ [32,33]. However, in plants, PI-PLC has only one pair of EF-hand domains [34]. In sorghum, PI-PLCs can be classified into two categories based on the presence or absence of EF-like domains: *SbPI-PLC1*, *SbPI-PLC2*, and *SbPI-PLC4* contain EF-like domains, while *SbPI-PLC3a*, *SbPI-PLC3b*, and *SbPI-PLC5* lack EF-like domains. As reported, the deletion of the EF-hand domain does not bind to Ca^2+^ [34,35], thereby attenuating PI-PLC activity [36,37,38]. However, it has been reported that the C2 domain can also bind to Ca^2+^ [32,39]. In *AtPI-PLC*, the EF-hand-like domain is deemed essential. As phospholipases responding to Ca^2+^ ion signals, the strength of the Ca^2+^ binding capability is directly associated with their functionality. All *SbPI-PLC* genes contain the C2 domain, suggesting that in sorghum, PI-PLC genes primarily bind to Ca^2+^ through the C2 domain, regulating cellular activities [7,40]. The EF-hand domain appears non-essential in SbPI-PLCs, and further investigation is required to elucidate its specific function.

We conducted a phylogenetic analysis of *PLC* gene in sorghum, rice [15], maize [16], and Arabidopsis [41], revealing a close arrangement of genes into two subgroups: *PI-PLC* and *NPC* (Figure 2). Gene structure is crucial for both function and regulatory mechanisms of expression. Variations in gene structure result from the gain or loss of exons and introns, including exon/intron gain/loss, exonization/pseudoexonization, and insertion/deletion [42]. This study identifies a common characteristic within the *PLC* gene family: *PI-PLC* gene structures are complex, whereas *NPC* gene structures are relatively simple. Research indicates that intron-poor genes often result from selective pressure in plants, correlating with a heightened response to abiotic stress. These findings also offer insights for future research on *PLC* genes [43]. *SbPLC4*, *SbPLC5*, and *SbNPC2* contain long introns, a unique structure also observed in maize (*ZmPLC2*, *ZmPLC4*), wheat (*TaPLC2-1A*, *TaPLC2-1D*, *TaNPC5-3A*), and rice (*OsNPC3*) but which is absent in *Arabidopsis* and tomato. In humans, longer introns are linked to functional roles, influencing gene expression regulation. However, the implications of these structures in plants require further investigation [44]. This suggests a high degree of homology within the same branch, indicating potential functional similarities. Disparities in the number of PI-PLC subgroup members are notable among different species, with six in sorghum, five in maize, four in rice, and nine in *Arabidopsis*. This implies a reduction in the PLC gene family during the evolutionary process in sorghum, with some functionally similar or non-essential genes diminishing over time. In the analysis of synteny with other plants, we found more conserved relationships with *OsPLCs* and fewer with *ZmPLCs* (Figure 4A–C). Among the six conserved relationships in maize, five were *ZmNPCs*, and only one was *ZmPLC*. In *Arabidopsis*, five out of six conserved relationships were *AtPLCs*, and in rice, eight out of thirteen were *OsPLCS*, with the remainder being *OsNPCs*. This suggests functional diversification in the evolution of *SbPLCs*, highlighting the need for further functional and in-depth genomic analyses to understand the evolutionary history of *SbPLCs* and their distinctions in signaling and growth development [45].

The analysis of *cis*-regulatory elements in gene promoters provides valuable insights into gene function [46]. Current research indicates a crucial correlation between *cis*-regulatory elements in the promoter region and the expression levels of specific genes. In the promoter regions of *SbPLCs*, multiple cis-regulatory elements involved in plant growth, hormone response, and abiotic/biotic stress were identified, suggesting a significant role for *SbPLCs* in sorghum’s adaptation to its environment (Figure 5). Among the identified cis-regulatory elements, MYB and MYC (linked to abiotic stress responses), along with ABRE (involved in hormone signaling), were present in all *SbPLCs*, suggesting their roles in mediating stress responses through ABA signaling [47,48]. This is consistent with findings in *AtPLC1* and *AtPLC3*, which are involved in ABA-regulated seed germination and seedling growth, where exogenous ABA inhibits *AtPI-PLC* activity [26,49]. TFs, proteins that regulate gene expression patterns and respond to biological signals within cells, play crucial roles in organisms [50]. In addition to the *cis*-elements, transcription factor binding site predictions identified Dof and ERF as having the most binding sites in *SbPLCs* (Figure 6). These transcription factors are known to play important roles in the ABA signaling pathway. For instance, *OsDofs* enhance rice’s tolerance to flooding during germination by responding to both GA and ABA [51], while *CmDOF12* and *CmDOF20* in chrysanthemum are also responsive to ABA signaling [52]. In *Arabidopsis*, *AtERF1* is negatively regulated by ABA, affecting its resistance to abiotic stress, and in cotton, *GhERF2*, *GhERF3*, and *GhERF6* respond to both ethylene and ABA signals [53,54]. In this study, qRT-PCR analysis demonstrated that *SbPLC* expression levels were upregulated under ABA treatment, further suggesting their role in sorghum’s adaptation to environmental stress (Figure 12A). These findings contribute to a deeper understanding of PLC’s involvement in ABA-mediated stress responses and highlight the broader regulatory mechanisms governing *SbPLCs* under abiotic stress.

In recent years, extensive research has confirmed the significant involvement of miRNAs in sorghum growth processes and their key roles in various stress responses through a whole-genome examination [55,56,57]. In this study, we identified 12 miRNAs from 9 families targeting 9 *SbPLCs* (Figure 7; Table S5). While no reports exist on miRNA-mediated regulation of *PLC* genes, previous studies have associated miR408 with stress responses in various plants, regulating plant stress responses [58]. In sorghum, sbi-miR408 is notably involved in Cd stress responses, playing a pivotal role in sorghum’s tolerance to Cd [59]. Furthermore, miR1432 plays a crucial role in disease resistance in rice, while sbi-miR1432 and sbi-miR6223 participate in sugar synthesis in sorghum leaves [60,61]. It has been documented that sbi-miR5566, sbi-miR5386, and sbi-miR5385 contribute to sorghum’s heat tolerance, with sbi-miR5386 and sbi-miR5385 also being involved in sorghum’s cell wall synthesis and growth [62,63,64]. MiR397, as an important miRNA, participates in various plant activities and regulates stresses such as drought, low temperature, and heavy metals. Additionally, sbi-miR397-5p is involved in sorghum Cd stress responses [59,65,66]. Particularly noteworthy is the lack of reports on the sbi-miR6223-5p, sbi-miR6229-3p, sbi-miR6232a-5p, and sbi-miR6234a-5p families of miRNAs. In summary, these studies suggest that miRNAs may regulate the transcriptional levels of *SbPLCs* under abiotic stress, thereby playing crucial roles under various stress conditions.

Through the analysis of *SbPLCs* expression patterns, it is evident that they manifest distinct expression in various tissues of sorghum. The expression levels of *SbPLCs* exhibit variations across different tissues and developmental stages, suggesting their potential involvement in the growth and development of sorghum (Figure 8). Previous studies have reported similar findings, such as the involvement of *AtNPC2* and *AtNPC6* in plant growth and development [67] and differential expression of *OsPLC* during developmental stages [15]. Notably, *SbNPC1* expression is detected in nearly all tissues and at elevated levels. In contrast, *OsNPC1* and *ZmNPC1*, closely related evolutionarily to *SbNPC1*, do not demonstrate a similar pattern, implying their pivotal role throughout the life activities of sorghum and necessitating further investigation [31]. qRT-PCR was employed to gauge the expression levels of stress-induced *SbPLCs*. *SbPLCs* expression is influenced by various stress conditions. During ABA treatment, nearly all *SbPLCs* exhibited changes in expression levels, revealing distinct patterns between aerial and underground tissues (Figure 12A). These findings support the idea that SbPLCs function as ABA signaling transduction factors. Notably, several *SbPLCs* (*SbPLC2*, *SbPLC3b*, *SbPLC5*, and *SbNPC3*) consistently showed an upregulation trend across all four stress treatments (Figure 12B–D). Additionally, pronounced tissue-specific changes in expression were observed between aerial and underground tissues under various stress conditions. For example, under NaCl stress, expression levels of these genes increased in the roots but decreased in the aerial parts. Conversely, under NaHCO_3_ stress, expression was upregulated in the aerial parts and downregulated in the roots. These findings suggest that different PLC family members uniquely contribute to stress adaptation mechanisms in sorghum. Similar responses have been observed in other plants, such as the enhancement of heat tolerance in rice by *AtPI-PLC9* [23], the negative regulation of salt tolerance in seedlings by *AtPI-PLC4* [22], and the improved cadmium tolerance in tobacco by *PsPI-PLC6* [68]. The specific functions of *SbPLCs* in sorghum await further investigation.

Gene co-expression networks have been utilized to screen gene sets or gene pairs involved in the same biological pathways or interactions [69,70]. Due to limited sample size and correlations between gene modules and tissues, WGCNA has been widely employed to identify co-expressed gene pairs in different plant tissues or experimental treatment samples [71]. Previous transcriptomic studies in sorghum primarily focused on WGCNA analysis of genes under individual treatments [4,72]. In this study, we employed the same WGCNA pipeline to construct gene co-expression networks of several key response modules to abiotic stress containing *SbPLCs* (Figure 10A): ABAR/magenta containing *SbPLC4*, NaHCO_3_L/yellow containing *SbPLC1*, and SALTR/black containing *SbPLC3a.* These three *SbPLCs* were not found in the same module or tissue sample, suggesting potential diverse functions of these genes in response to abiotic stress. To further investigate the potential correlations among these three genes, PPI analysis was conducted on these three genes and their co-expressed genes, establishing a PPI network (Figure 10B). The analysis revealed direct interactions among these three genes, and their co-expressed genes also exhibited interactions with each other, forming a protein–protein interaction network. In wheat, PAL has been shown to affect the activity of PLC proteins, suggesting a potential influence of *SbPAL* on SbPLC activity [73]. Additionally, key transcription factors, such as SbARF6 and SbSCL1, may also impact the activity of *SbPLCs*. The results of the GO enrichment analysis indicate that *SbPLCs* and their significantly co-expressed genes are enriched in responses to abiotic stress and transcription processes (Figure 10). Therefore, our findings suggest that *SbPLCs* and their co-expressed genes exhibit specific expression patterns under abiotic stress, playing crucial roles in sorghum’s response to non-biological stressors.

## 4. Materials and Methods

### 4.1. Identifying Members of the PLC Gene Family in Sorghum

To identify members of the *PLC* gene family in sorghum, genome sequence files and annotations were downloaded from the Ensembl plants (https://ensembl.gramene.org/index.html (accessed on 23 May 2024)) [74]. Two methods were employed for *PLC* gene family identification: (1) To identify PLC proteins, Hidden Markov Model (HMM) files corresponding to the characteristic protein domains of PLCs were downloaded from the PFAM protein family database (https://www.ebi.ac.uk/interpro/download/Pfam/ (accessed on 23 May 2024)). The HMMs for the PI-PLC-X (PF00388), PI-PLC-Y (PF00387), PI-PLC-C2 (PF00168), and the phosphoinositide phospholipase C (NPC) phosphoesterase domain (PF04185) were utilized as queries in the HMM-based local search, setting the E-value threshold to <0.01. (2) Using the protein sequences of identified *Arabidopsis thaliana* AtPLCs from The *Arabidopsis* Information Resource (https://www.arabidopsis.org/ (accessed on 23 May 2024)), the sorghum genome was subjected to identification using the BLASTP (protein blast) method with an E-value cutoff of <0.01. Subsequently, the SMART database (https://smart.embl.de/ (accessed on 23 May 2024)) and NCBI CDD (https://www.ncbi.nlm.nih.gov/Structure/bwrpsb/bwrpsb.cgi (accessed on 23 May 2024)) were employed to further confirm the protein domains of the PLC family. Sequences that were redundant, duplicated, or had incomplete annotations were excluded.

### 4.2. Feature Analysis and Phylogenetic Tree Analysis of SbPLCs Protein Sequences

For the identified sorghum PLCs proteins, their full-length amino acid sequences, CDS length, molecular weight (MW), isoelectric point (PI), and other physicochemical properties were analyzed using the online tool ExPASy-ProtParam (https://web.expasy.org/protparam/ (accessed on 24 May 2024)). A subcellular localization analysis was conducted using SignalP Server (http://www.cbs.dtu.dk/services/SignalP/ (accessed on 24 May 2024)) and WoLF PSORT Prediction (https://wolfpsort.hgc.jp/ (accessed on 24 May 2024)).

Protein sequences from the PLC families of *Sorghum bicolor*, *Arabidopsis thaliana*, *Zea mays*, and *Oryza sativa* were compared using the ClustalW method. The alignment results were used to construct a phylogenetic tree with MEGA 11.0 software [75], employing the maximum likelihood method (ML) with a bootstrap value set to 1000 for validation. The final evolutionary tree was refined using evolview (http://www.evolgenius.info/evolview/#/treeview (accessed on 8 June 2024)).

### 4.3. Exon–Intron Structure and Conserved Motif Sequence Analysis of the Sorghum PLC Gene Family Members

For predicting conserved elements in *SbPLCs*, the online tool MEME (https://meme-suite.org/meme/tools/meme (accessed on 8 June 2024)) was utilized with the configuration of 10 motifs and default parameters. The conserved motifs in *SbPLCs* gene family members were visualized using TBtools [76]. Genomic DNA sequences and CDS sequences of *SbPLCs* were extracted from the sorghum genome database. Comparative analysis of CDS and genomic sequences was conducted using the Gene Structure Display Server (GSDS, http://gsds.cbi.pku.edu.cn/ (accessed on 8 June 2024)), illustrating exon–intron structures. Images were created using TBtools.

### 4.4. Chromosomal Localization, Gene Duplications Analysis, and Substitution Rate Analysis of SbPLCs

To obtain and analyze chromosomal framework information and the positional details of SbPLCs on different chromosomes, the Tbtools software v2.0 was employed. Gene density distribution analysis and visualization were conducted using the Gene Density Distribution tool within TBtools. The Circos plot was generated using the Fasta Start tool in TBtools to obtain chromosomal framework information, the Table Row Extract or Fitter tools to acquire gene density files, and the MCScan X tool to retrieve collinearity information.

Genomic data for *A. thaliana*, *Z. mays*, and *O. sativa* were downloaded from Ensembl Plants (https://ensembl.gramene.org/index.html (accessed on 8 June 2024)). The ONE STEP MCScan X functionality in Tbtools was utilized for collinearity analysis among sorghum and the three species. Non-synonymous (Ka) and synonymous (Ks) substitution values were calculated using MEGA software.

### 4.5. Analysis of Cis-Acting Elements in SbPLCs’ Promoters

To analyze the cis-acting elements in the promoters of *SbPLCs*, we extracted the sequences upstream of the initiation codon (2k bp) from the sorghum genome. Subsequently, the promoter sequences of each gene were analyzed using the PlantCARE website (http://bioinformatics.psb.ugent.be/webtools/plantcare/html (accessed on 8 June 2024)). Heatmaps were generated using the ggplot2 package in R.

### 4.6. Predicting Transcription Factors Regulating SbPLCs

To identify potential transcription factors (TFs) regulating *SbPLCs*, we analyzed the 2000 bp promoter regions of each SbPLCs using the PlantRegMap tool (http://plantregmap.gao-lab.org/ (accessed on 8 June 2024)). TFs meeting the criteria of q-value < 0.05 were retained, and a TFs abundance heatmap was generated using TBtools.

### 4.7. Prediction of Putative miRNA Targeting SbPLCs

The coding sequences (CDS) of *SbPLCs* were used to identify potential miRNA targets on the psRNATarget database (https://www.zhaolab.org/psRNATarget/home (accessed on 8 June 2024)) using default parameters.

### 4.8. Expression Pattern Analysis of SbPLCs in Different Tissues

The transcriptional data of sorghum obtained from the European Molecular Biology Laboratory’s (EMBL’s) European Bioinformatics Institute database under accession numbers E-MTAB-5956 [77], E-MTAB-4273 [78], E-GEOD-98817 [79], and E-CURD-25 [80] were analyzed. The identified *SbPLCs* were then input into SorghumFDB (http://structuralbiology.cau.edu.cn/sorghum/index.html (accessed on 10 June 2024)) to obtain tissue-specific expression data. Subsequently, this data was imported into TBtools for heatmap generation. SorghumFDB functions as a dedicated sorghum database, facilitating the visualization of expression data, clustering, and the construction of correlation networks.

### 4.9. Expression Profiles of SbPLCs Under Abiotic Stress

Utilizing transcriptome data treated with ABA (20 μM), 20% PEG-6000, 120 mM NaCl [81], and 150 mM NaHCO_3_ [70], the expression patterns of *SbPLCs* under abiotic stress were investigated. The accession numbers for the datasets are PRJNA143783, PRJNA395348, PRJNA816817, and PRJNA1000939. A heatmap analysis of expression patterns was conducted using the logarithm of gene expression fold change (log_2_FC) compared to control tissues, and the heatmaps were generated using Tbtools.

### 4.10. Construction of the Co-Expression Network and Protein–Protein Interaction Network Analysis

Differentially expressed genes filtered from the above datasets including leaf and root tissues, were normalized as RPKM > 10 and selected for WGCNA using the R software package (version 3.2.2) to construct their co-expression network. Firstly, a Pearson correlation matrix of all gene pairs was generated based on the measured samples. Subsequently, an adjacency matrix representing the strength of connections between genes was constructed. The adjacency matrix was calculated by raising the measure of co-expression to a chosen soft thresholding power β = 7 (Figure S6), aiming to achieve an approximate scale-free topology with a fitting index of around 0.80. Based on the adjacency matrix, a topological overlap matrix (TOM) was computed to measure the interconnectedness within the network. Hierarchical clustering was employed to cluster genes into different modules based on the topological overlap matrix. Genes belonging to different modules were assigned different colors for visualization, with grey indicating genes not assigned to any module. To identify modules associated with ABA, salt, drought, and NaHCO_3_ stress, characteristic genes of modules were correlated with ABA, salt, drought, and NaHCO_3_ using Pearson correlation coefficients. Modules with a *p*-value < 0.05 were identified as stress-related modules. Modules containing SbPLCs were selected for further analysis, with a correlation coefficient ≥ 0.8 and *p*-value ≤ 0.05. Subsequently, the co-expressed genes of *SbPLCs* within the selected modules were queried for protein–protein interactions (PPI) on the STRING database (https://version-12-0.string-db.org/ (accessed on 8 June 2024)). Finally, the identified co-expressed genes and interacting proteins were visualized using Cytoscape v3.7.1 [82].

### 4.11. GO and KEGG Analysis of Genes in Co-Expression Modules

To further characterize the biological functions of the stress-related modules of interest, we performed an enrichment analysis of Gene Ontology (GO) and Kyoto Encyclopedia of Genes and Genomes (KEGG) pathways using the Cluster Profiler package. KEGG pathways with *p*-values < 0.05 were considered significantly enriched.

### 4.12. RNA Extraction and Quantitative Expression Analysis Under Different Abiotic Stresses Using qRT-PCR

To disinfect sorghum seeds, they were treated with a 2% sodium hypochlorite solution for 8 min, followed by three rinses with distilled water. Subsequently, all seeds were placed on moist filter paper and cultivated in a growth chamber (23 °C, 16 h light/8 h dark) until sprouts of 0.3–0.5 cm appeared. Seedlings were then transplanted into boxes and grown in 1/2-strength medium. Treatments with 150 mM NaCl, 100 μM ABA, 150 mM NaHCO_3_, and 20% PEG were applied, and samples were collected at 6, 12, and 24 h after treatment. The materials were flash-frozen in liquid nitrogen and stored at −80 °C for subsequent experiments. RNA extraction and expression analyses were performed using quantitative real-time reverse transcription-polymerase chain reaction (qRT-PCR). The expression profiles of *SbPLCs* under various abiotic stresses were analyzed using specific primers (Table S1) through qRT-PCR of which analysis was performed using SYBR Premix Ex Taq™ (TaKaRa, Shuzo, Otsu, Japan). Three biological replicates for each sample and three technical replicates for each biological replicate were used for the analysis. *SbACT1* (LOC110436378) served as the internal reference gene, and the 2^−ΔΔCT^ method was used to calculate the relative expression values [83].

### 4.13. Statistical Analysis

Statistical analysis was performed using SPSS software 30.0.0. ANOVA (*p* < 0.05) was chosen for the statistical analyses. Data are presented as mean ± SD of at least three independent biological replicates.

## 5. Conclusions

In summary, our study of the *SbPLC* gene family identified 11 genes, including *SbPI-PLCs* and *SbNPCs*, enhancing our understanding of lipid signaling regulators in *sorghum*. Promoter analysis, transcription factors, and miRNAs offer new insights into the transcriptional regulation of *SbPLCs*. The expression patterns observed across various tissues and developmental stages underscore the dynamic role of *SbPLCs* in essential physiological processes. The qRT-PCR results indicate that most *SbPLCs* members exhibit differential expression levels under various stress conditions, revealing specific and overlapping patterns. WGCNA analysis identified *SbPLC1*, *SbPLC3a*, and *SbPLC4* as core genes related to abiotic stress. These findings provide valuable insights for further research on the roles of *SbPLCs* in plant development and responses to environmental stress.

## Figures and Tables

**Table 1:** General information on *PLC* genes in *Sorghum bicolor*.

Gene Name	Gene ID	ORF (bp)	AA	MW (KD)	pI	TNA	GRAVY	Sub-Localization Prediction
*SbPI-PLC1*	SORBI_3002G430500	1824	607	69.36	6.07	9696	−0.593	Cell membrane
*SbPI-PLC2*	SORBI_3001G408001	1884	627	70.45	6.06	9872	−0.415	Cell membrane
*SbPI-PLC3a*	SORBI_3005G002000	1839	612	68.66	6.62	9602	−0.469	Cell membrane
*SbPI-PLC3b*	SORBI_3008G002000	1833	610	68.56	6.62	9590	−0.457	Chloroplast
*SbPI-PLC4*	SORBI_3009G027800	1809	602	67.5	5.93	9420	−0.446	Mitochondrial
*SbPI-PLC5*	SORBI_3005G018900	2037	678	77.76	7.13	10863	−0.597	Cytoplasmic
*SbNPC1*	SORBI_3001G029200	1629	542	60.58	7.82	8497	−0.338	Chloroplast
*SbNPC2*	SORBI_3003G430100	1572	523	57.67	6.02	8046	−0.335	Chloroplast
*SbNPC3*	SORBI_3001G008600	1623	540	59.39	6.03	8257	−0.38	Chloroplast
*SbNPC4*	SORBI_3005G170100	1533	510	56.75	5.76	7864	−0.355	Chloroplast
*SbNPC5*	SORBI_3003G107900	1809	602	63.43	9.17	8909	−0.175	Mitochondrial

## Data Availability

The data presented in this study are available in the European Molecular Biology Laboratory’s (EMBL’s) European Bioinformatics Institute database at [https://www.embl.org/ (accessed on 10 June 2024)], reference numbers E-MTAB-5956, E-MTAB-4273, E-GEOD-98817, and E-CURD-25.

## Supplementary Materials

The following supporting information can be downloaded at https://www.mdpi.com/article/10.3390/plants13212976/s1, Figure S1: Conserved Domain of SbPLC Proteins. Figure S2: The *SbPLCs* motifs.; Table S1: The list primer was used for gene expression analysis by qRT-PCR; Table S2: The Ka/Ks ratio of the *SbPLC* gene family; Table S3: Collinear gene pairs with *SbPLCs* in other species; Table S4: Predicted transcription factors; Table S5: Information of predicted miRNA targeting *SbPLCs.*
