# Supplementary material for: Genome-Wide Identification and Analysis of Phospholipase C Gene Family Reveals Orthologs, Co-Expression Networks, and Expression Profiling Under Abiotic Stress in Sorghum bicolor
Source: Plants (Basel). 2024 Oct 24;13(21):2976. doi: 10.3390/plants13212976 (PMC11547881; doi:10.3390/plants13212976)
Supplement: Supplementary file 1 [file plants-13-02976-s001.zip › supplementary.pdf]

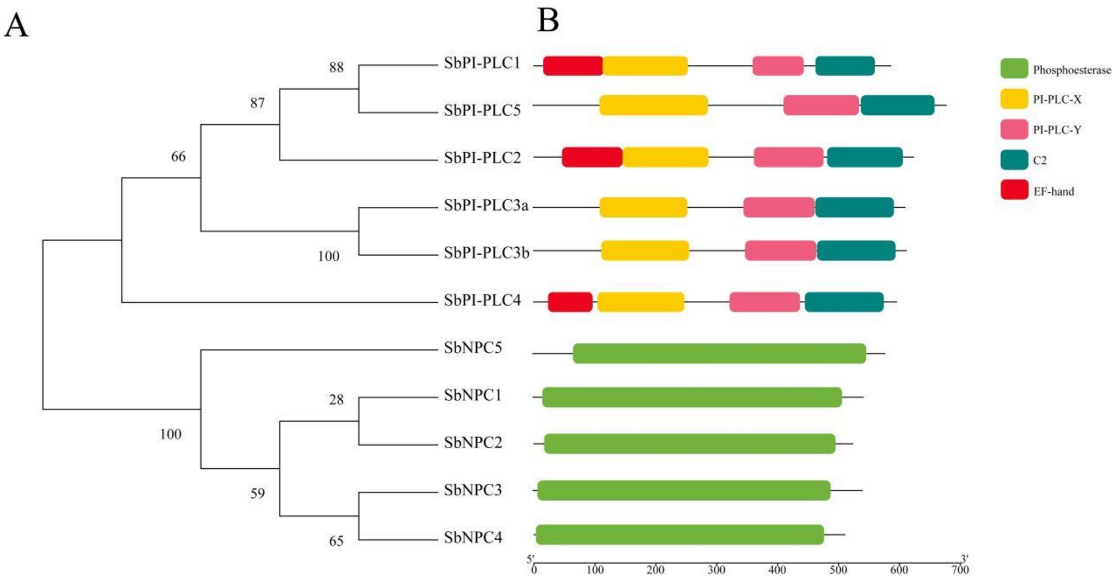


**Fig. S1 Conserved Domain of SbPLC Proteins.** (A) Phylogenetic tree of SbPLCs. The tree was generated using MEGA7 through the Maximum Likelihood method based on the protein sequences of SbPLCs. (B) Conserved Domain of SbPLC Proteins.

motif 1
motif 2
motif 3
motif 4
motif 5
motif 6
motif 7
motif 8
motif 9
motif 10
motif 11
motif 12
motif 13

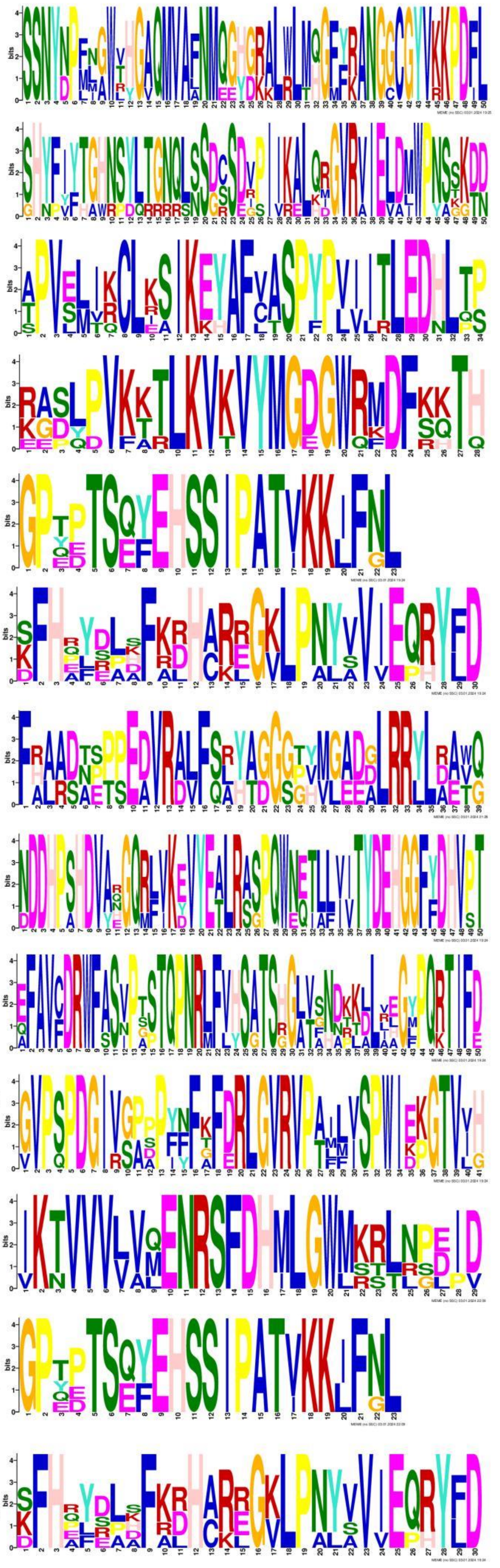

**Fig. S2** The *SbPLCs* motifs. The overall height of each stack indicates theconservation of the sequence at that position, whereas the height of letters within eachstack represents the relative frequency of the corresponding amino acid.
